# Implementation of a Standardized Surgical Operative Note: A Clinical Audit at Atbara Teaching Hospital

**DOI:** 10.7759/cureus.88685

**Published:** 2025-07-24

**Authors:** Abubakr Muhammed, Mustafa Awad, Mawada Taha, Shorouq Mohammed Ali, Moaid Abdallah Mohamed Alameen, Zainab Hussein Musa Mohamed, Mohamed Kamalaldein Hamad Mohamednour, Israa Isam Mohamed, Ahmed Shakir Ali Yousif, Ola Atif Mubarak Abdullah, Muayad Mubarak Mohamed Ali, Ashraf Mohamed Suliman Mohamed, Ahmed Yahia Mahadi Babiker, Abdalgader Omer Saeed Majzoub, Mohammed Abd Elgaioum Sailh Omer, Abdulmonem Mohamed Ahmed Mohajer, Mohammed Salaheldin Elsheikh Ahmed, Tagwa Fadalla, Shereen Abdelmoniem Yousif Abdulhadi, Mustafa Mohamed

**Affiliations:** 1 Surgery, University of Gezira, Madani, SDN; 2 Internal Medicine, Sudan Medical Specialization Board, Khartoum, SDN; 3 General Surgery, The National Ribat University, Khartoum, SDN; 4 General Surgery, Wad Madani University for Science and Technology, Wad Madani, SDN; 5 General Surgery, Atbara Teaching Hospital, Atbara, SDN; 6 Dental Public Health, Sudan Medical Specialization Board, Khartoum, SDN; 7 Surgery, Federal Ministry of Health, Khartoum, SDN; 8 Urology, Gezira Hospital for Renal Diseases and Surgery, Wad Madani, SDN; 9 General Surgery, Ahfad University, Omdurman, SDN

**Keywords:** clinical audit, documentation, low-resource settings, quality improvement projects, surgical operation notes

## Abstract

Background and aim: High-quality surgical operation notes are essential for patient safety, clinical communication, and medicolegal accountability. In low-resource settings like Sudan, inconsistent and incomplete documentation remains a challenge. This study aimed to evaluate and improve the quality of surgical operation notes at the Atbara Teaching Hospital using a closed-loop clinical audit.

Methods: A two-cycle audit was conducted using a standardized checklist based on the guidelines of the Royal College of Surgeons of England. Fifty surgical notes were reviewed in each cycle. Following the baseline assessment, interventions including the implementation of a structured proforma and staff education sessions were introduced. A re-audit was then performed to assess the impact of these measures.

Results: Significant improvements were observed across most documentation parameters. Patient identification improved from 2% to 100% (p<0.0001), preoperative diagnosis from 8% to 93.5% (p<0.0001), and postoperative care instructions from 0% to 76.1% (p<0.0001). Documentation of anticipated blood loss, antibiotic prophylaxis, and closure techniques also showed statistically significant enhancements. However, gaps remained in areas, such as complication reporting and anesthetist details.

Conclusion: Structured interventions, including standardized templates and targeted training, substantially improved the completeness and quality of surgical operation notes. Ongoing audits, reinforced education, and potential integration of electronic records are recommended to sustain and build upon these improvements, particularly in resource-limited settings.

## Introduction

Surgical operation notes are comprehensive records detailing each step of the perioperative journey, from preoperative diagnosis and intraoperative findings to postoperative care instructions. They serve as an essential communication tool among the surgical team, nursing staff, anesthetists, and future care providers. Beyond patient care, these notes are vital for medicolegal protection, billing, and institutional audits. The Royal College of Surgeons has underscored that operation notes are “arguably the single most important document in all of surgery” [1].

High-quality operative documentation is synonymous with surgical professionalism. Precise and structured notes ensure that subsequent clinicians can understand the specifics of the surgery, anticipate potential complications, and deliver safe postoperative care. Conversely, missing details, illegible text, or absent postoperative plans have been associated with delayed interventions, compromised patient outcomes, and medicolegal risks [2].

Auditing surgical operation notes involves systematically reviewing documentation against established guidelines to identify omissions, inconsistencies, or errors. This process helps to pinpoint which elements, such as time of operation, patient position, anesthetist’s name, and details of bleeding, are frequently underreported or improperly recorded. Studies have consistently shown that incorporating audits, education sessions, and structured proformas leads to significant improvements in documentation completeness and consistency, often resulting in compliance rates exceeding 80% across all evaluated parameters [3].

Auditing operative note quality is a proven intervention to bridge documentation gaps. Prospective audit cycles featuring baseline measurements, interventions, such as standardized templates and staff education, and subsequent reassessment have consistently led to notable improvements in documentation, often increasing compliance by 30-40% or more [4,5]. In this audit, the primary goal was not to reach a fixed threshold, such as 80%, but rather to demonstrate meaningful improvement in adherence to the Royal College of Surgeons (RCS) documentation standards. Such improvements are valuable in enhancing communication, ensuring medicolegal safety, and promoting consistency in surgical practice. Closed-loop audit approaches further reinforce good practice and help foster long-term cultural change in clinical documentation.

In Sudan, hospitals, such as Dongola and Port Sudan Teaching Hospitals, face contextual challenges, including heavy caseloads, limited digital infrastructure, and constrained resources. A three-month audit at Dongola showed baseline compliance of 50.3%, which increased to 71.9% after implementing a structured proforma and training program [6]. Similarly, Port Sudan’s audit demonstrated improvement from 51.9% to 82.1% following the standardized note formats and educational sessions [7].

Building on this evidence, the quality improvement process conducted at Atbara Teaching Hospital in August 2024 aimed to audit surgical operation notes, implement targeted interventions (structured templates and training), and re-evaluate documentation quality in a closed-loop process. The objective was to align local practice with international standards, enhance medicolegal robustness, and ultimately promote patient safety in a resource-limited context.

## Materials and methods

This quality improvement audit was conducted at Atbara Teaching Hospital in two cycles. The first audit cycle involved retrospective review of surgical operation notes documented between August 27, 2024, and November 30, 2024, to identify documentation gaps. Following this, targeted interventions, including educational sessions and the introduction of a standardized proforma, were implemented from December 1, 2024, to March 15, 2025. This extended intervention period allowed for phased implementation and adequate staff engagement to ensure sustainable improvements. The second audit cycle prospectively assessed operation notes from March 16, 2025, to May 2, 2025, to evaluate the impact of the interventions on documentation quality.

The purpose of this audit was to back up more accurate surgical documentation by encouraging uniformity, precision, and legal sufficiency in the recording of operation notes. Inconsistent or partial surgical documentation poses a threat to patient safety and continuity of treatment in resource-constrained situations like Sudan. An effective way to find systemic flaws and make low-cost, practical changes to clinical communication and accountability is to conduct systematic audits.

The project's overarching goal was to help foster a culture of continuous improvement in the institution, going beyond its specific aims. Clinical audits provide a repeatable method for finding areas of practice that need improvement, implementing specific changes, and then periodically evaluating the results. When these audits become normal operating procedure, they improve surgical service quality by reinforcing clinical governance and ensuring adherence to changing best practices.

Data collection

The data collection process was guided by a modified version of the Royal College of Surgeons of England (RCS) operative note documentation standards [8]. The original checklist was reviewed and adapted to the local context at Atbara Teaching Hospital to reflect regional practices and resources. This adaptation involved adding clarifying descriptions for certain parameters to enhance consistency and removing elements that were not relevant to the setting (e.g., electronic documentation requirements). The final modified checklist comprised 18 key parameters covering essential aspects of operative documentation, including preoperative details, intraoperative findings, safety measures, and postoperative instructions (Table 1). The checklist was reviewed and approved by the hospital’s surgical quality assurance committee prior to use.

**Table 1:** Checklist of essential parameters for surgical operation notes. This checklist was developed in accordance with the documentation standards set by the Royal College of Surgeons (RCS) of England. It includes the core parameters recommended for high-quality operative note writing, ensuring completeness, clarity, and medicolegal reliability. The checklist was used to evaluate the quality and completeness of surgical notes during the audit cycles. Record Your Work Clearly, Accurately, and Legibly (2024) [8]. LMWH: low molecular weight heparin; DVT: deep vein thrombosis

Parameters	Description
Date and time	Record the exact date and time of the procedure.
Type of operation	Specify the procedure performed (e.g., appendectomy).
Name of operator and assistant	Include full names of the lead surgeon and assisting staff.
Name of anesthetist	Document the anesthetist responsible for the case.
Operative procedure carried out	Describe the procedure actually performed, including modifications, if any.
Incision type	State the location and type of incision made.
Operative diagnosis	Provide the final intraoperative diagnosis.
Operative findings	Note key intraoperative findings including pathology and anatomical details.
Problems/complications	Mention any intraoperative issues or complications encountered.
Any extra procedures and why performed	Detail any additional procedures done and the reasons for them.
Details of tissue removed, added, or altered	Describe any excised or grafted tissues and anatomical alterations.
Identification of prostheses/material used	Provide details of any implants or foreign materials (e.g., mesh, plates).
Details of closure technique	Describe how the wound was closed (e.g., sutures, staples, layers).
Anticipated blood loss	Estimate the amount of blood lost during the procedure.
Prophylactic antibiotics	Record type, dose, and timing of antibiotics given pre-/intraoperatively.
Deep vein thrombosis (DVT) prophylaxis	State whether DVT prophylaxis was used and its form (e.g., LMWH, compression).
Postoperative care instructions	Include postoperative orders, follow-up, and any specific care instructions.
Surgeon’s signature	Surgeon must sign the note to validate the documentation.

All consecutive operative notes for both elective and emergency surgical procedures performed in the general surgery department between January 1, 2025, and March 31, 2025, were reviewed. Notes were excluded if they were incomplete due to ongoing procedures or if the surgery was conducted outside the department. In total, 50 operative notes were included in the first audit cycle.

Data were collected by two surgical residents and one senior medical officer who underwent a standardization session to ensure consistent interpretation of the checklist. A pilot review of 10 operative notes was carried out before the start of formal data collection to enhance clarity and improve inter-rater agreement. Each operative note was assessed within 24 hours of surgery to minimize recall bias. Compliance with each parameter was recorded as yes (documented) or no (not documented) using a Google Form specifically designed for this audit, which automatically populated responses into an electronic spreadsheet for analysis.

When documentation was ambiguous or unclear, the data collectors sought clarification from the operative team whenever possible. If clarification could not be obtained, the item was marked as “not documented.” No retrospective modifications of operative notes were allowed during the review process. To ensure confidentiality, all patient identifiers were removed before data entry, and the dataset was stored on a password-protected computer accessible only to the audit team.

Study population and setting

Atbara Teaching Hospital is a big referral center serving a diverse population in a resource-limited area of Sudan. Surgeons operating on adults (those aged 18 years and over) at this hospital were the subject of this audit. A wide range of surgical specialties is available at this facility, which treats both routine and urgent patients.

All adult patients who had surgery during the audit periods had their operation notes included. If the surgery took place outside of the hospital, if the paperwork was inadequate to do an evaluation, or if the patient's treatment was cancelled, then the notes would not be included. The demographics of the study cohort, including age, sex, and underlying comorbidities, were representative of the average surgical workload in this environment. Insights into the local healthcare standard of care were derived from this demographic profile, which enabled a thorough assessment of documentation quality across several surgical procedures.

Sampling technique

All eligible surgical operation notes were included throughout the audit periods using a comprehensive coverage sampling technique. One hundred operation notes were analyzed for the research; 50 notes were examined in each of the two audit cycles. The audit took place over the course of six weeks as follows: two weeks for gathering baseline data, two weeks for orientation and intervention implementation, and two weeks for conducting follow-up audits.

We ensured a representative sample of surgical operations across all specialties and case difficulties by meticulously collecting data from medical records in the hospital's archive and surgical wards. In order to provide a strong picture of documenting procedures throughout the research period, our exhaustive sampling strategy made sure that every operation note that satisfied the inclusion criteria was evaluated.

The time frames - August 27 to November 30, 2024 (first cycle), December 1, 2024, to March 15, 2025 (intervention period), and March 16 to May 2, 2025 (second cycle) - were selected to allow sufficient time for both baseline evaluation and postintervention assessment. Both cycles' complete coverage sampling methods were consistent and comparable, which lent credence to the audit's results and the quality improvement program’s efficacy.

Statistical analysis and data

IBM SPSS Statistics version 25 (Armonk, NY: IBM Corp.) was used for data entry and analysis. The dataset comprised all eligible operative notes within the defined study periods, and no sample size calculation was required as this was a complete audit of consecutive cases. Descriptive statistics were employed to summarize the data as follows: categorical variables were expressed as frequencies and percentages, while continuous variables were reported as means with standard deviations where applicable.

The primary outcome was compliance with documentation standards across two audit cycles. Because the outcome variables represented categorical data (presence or absence of documentation for each parameter), the chi-square test was applied to compare compliance rates between the preintervention and postintervention cycles. This test is appropriate for independent categorical variables. Before analysis, the assumptions of the chi-square test were checked: all expected cell counts exceeded five, and the data represented independent observations.

Missing data were handled by pairwise deletion, meaning that any parameter not documented in the operative note was recorded as “non-compliant” rather than excluded from analysis, ensuring completeness and avoiding bias. A p<0.05 was considered statistically significant for all comparisons.

Audit cycles

Initial Phase: Initial Evaluation

The first audit cycle was carried out from August 27, 2024, to November 30, 2024, to determine the current level of compliance with the documentation criteria for surgical operation notes. Notable gaps in note accuracy and completeness were uncovered during this baseline evaluation. These gaps included information about the date and time of the surgery, the identities of the operating personnel, intra-operative results, and post-operative instructions, among other things. These voids highlighted the need for focused initiatives to strengthen medicolegal robustness, enhance therapeutic communication, and improve documentation quality.

Targeted Strategy Implementation: The Intervention Phase

A number of planned initiatives were put in place from December 1, 2024, to March 15, 2025, to fix the problems found during the baseline assessment. As part of these efforts, a standardized operation note form was created and distributed with the goal of encouraging thorough documentation. At the same time, educational seminars and training sessions were organized for the surgical team, with an emphasis on the significance of complete documentation, legal factors, and professional standards. To keep doctors interested and engaged, the curriculum mixed lecture-style lectures with more hands-on case discussions. The surgical wards and operating theaters were designed with visual reminders, such as posters, that summarize important documentation aspects. Changes to processes, such as who is responsible for completing notes and holding regular feedback meetings, were also introduced to encourage responsibility. Plans were created for regular re-audits to ensure compliance and promote continual improvement, as well as for continuing education to bolster sustainability.

Phase Two: Assessing Outcomes Following Intervention

An evaluation of these measures’ effects on the quality of operation note documentation was carried out in the second audit cycle, which followed the intervention period, from March 16, 2025, to May 2, 2025. Compliance rates for all main documentation criteria showed significant increases in the results, with several items over 80% complete. The standardized templates and training programs were successful in addressing documentation gaps, and the increased knowledge among personnel contributed to this positive change. The Atbara Teaching Hospital’s surgical services saw an uptick in patient safety and a general improvement in medicolegal documentation standards thanks to the cyclical audit process, which also helped to uncover and fix practice gaps.

## Results

The results from the second cycle demonstrated notable improvements in documentation practices compared to the first cycle (Table 2). Patient identification was documented in only one (2%) case during the first cycle, compared to 50 (100.0%) in the second cycle (p<0.0001). Documentation of the date and time of the procedure improved from 28 (56%) to 49 (97.8%) (p<0.0001), while the type of the procedure was recorded in eight (16%) in the first cycle and 50 (100.0%) in the second (p<0.0001).

**Table 2:** Comparison of surgical operation note documentation parameters before and after intervention based on RCS guidelines. This table presents a comparative analysis of documentation compliance for key surgical operative note parameters between the first and second audit cycles, each comprising 50 surgical cases. The checklist used was derived from the Royal College of Surgeons (RCS) guidelines. The "improvement" column represents the percentage point increase in compliance between the two cycles. Statistical significance was evaluated using the chi-square (χ²) test. A p<0.05 was considered statistically significant. Notable improvements were observed in several critical parameters, indicating the effectiveness of structured interventions, such as standardized proformas and staff training. DVT: deep vein thrombosis

Parameters	First cycle (n=50)	Second cycle (n=50)	Improvement	Chi-square (χ²)	p-Value
Patient identification	1 (2%)	50 (100.0%)	98.0%	92.20	<0.0001
Date and time	28 (56%)	49 (97.8%)	41.8%	22.59	<0.0001
Type of procedure	8 (16%)	50 (100.0%)	84.0%	69.01	<0.0001
Surgical team information	38 (76%)	49 (97.8%)	21.8%	8.84	0.0029
Anesthetist information	29 (58%)	40 (80.4%)	22.4%	4.68	0.0306
Performed surgical procedure name	44 (88%)	50 (100.0%)	12.0%	4.43	0.0353
Type of incision	32 (64%)	42 (84.8%)	20.8%	4.21	0.0402
Preoperative diagnosis	4 (8%)	47 (93.5%)	85.5%	70.59	<0.0001
Operative findings	39 (78%)	46 (91.3%)	13.3%	2.82	0.0929
Complication encountered	26 (52%)	18 (37.0%)	-15.0%	1.99	0.1585
Extra procedures and rationale	10 (20%)	11 (21.7%)	1.7%	0.00	1.0000
Tissue details stated	27 (54%)	38 (76.1%)	22.1%	4.40	0.0360
Closure technique	23 (46%)	41 (82.6%)	36.6%	12.54	0.0004
Anticipated blood loss	5 (10%)	24 (47.8%)	37.8%	15.74	0.0001
DVT prophylaxis (if applicable)	0 (0%)	10 (19.6%)	19.6%	9.00	0.0027
Antibiotics prophylaxis	7 (14%)	32 (63.0%)	49.0%	24.21	<0.0001
Identification of prosthesis used	7 (14%)	13 (26.1%)	12.1%	1.56	0.2113
Postoperative care instructions	0 (0%)	38 (76.1%)	76.1%	58.11	<0.0001
Surgeon’s signature	20 (40%)	32 (63.0%)	23.0%	4.85	0.0277

Surgical team information was reported in 38 (76%) of first-cycle cases and 49 (97.8%) in the second (p=0.0029). Anesthetist information increased from 29 (58%) to 40 (80.4%) (p=0.0306), and the performed surgical procedure name was recorded in 44 (88%) in the first cycle and 50 (100.0%) in the second (p=0.0353). The type of incision documentation improved from 32 (64%) to 42 (84.8%) (p=0.0402), and a major improvement was seen in preoperative diagnosis documentation, increasing from four (8%) to 47 (93.5%) (p<0.0001).

Operative findings were recorded in 39 (78%) of first-cycle cases and 46 (91.3%) in the second, with no statistically significant difference (p=0.0929). Documentation of complications encountered decreased from 26 (52%) to 18 (37.0%) cases (p=0.1585), while extra procedures and rationale remained nearly unchanged at 10 (20%) and 11 (21.7%) cases, respectively (p=1.0000). Tissue details stated improved from 27 (54%) to 38 (76.1%) cases (p=0.0360), and closure technique documentation increased from 23 (46%) to 41 (82.6%) cases (p=0.0004).

Additional documentation aspects also showed substantial improvement. Anticipated blood loss was recorded in five (10%) and 24 (47.8%) cases in the first and second cycles, respectively (p=0.0001), and DVT prophylaxis was newly documented in 10 (19.6%) cases in the second cycle (p=0.0027). Antibiotic prophylaxis documentation increased from seven (14%) to 32 (63.0%) cases (p<0.0001), and postoperative care instructions rose from 0 (0%) to 38 (76.1%) cases (p<0.0001). Identification of prosthesis used improved modestly from seven (14%) to 13 (26.1%) cases, though not statistically significant (p=0.2113). Finally, the surgeon’s signature was present in 20 (40%) cases in the first cycle and 32 (63.0%) cases in the second (p=0.0277).

## Discussion

The results of this audit demonstrate a significant improvement in the quality of surgical operative documentation following structured interventions, consistent with trends reported both regionally and globally. As shown in Table 2, compliance with key documentation elements increased markedly between the first and second audit cycles. Parameters, such as patient identification (one {2%}-50 {100%}), type of procedure (eight {16%}-50 {100%}), and preoperative diagnosis (four {8%}-47 {93.5%}) showed statistically significant enhancements (p<0.0001), reflecting heightened attention to essential surgical details. It is important to note that the initially low rate of patient identification documentation reflects local practice, where patient identifiers are traditionally recorded on the patient file covers rather than within the operative notes themselves.

This contextual factor is important when interpreting the audit results, as it reflects differences in documentation practices that may exist between institutions. Awareness of such local practices helps provide a clearer understanding of the findings and their implications. These findings align with those reported by Port Sudan Teaching Hospital, where compliance increased from 51.9% to 82.1% following the implementation of an improved proforma and focused staff education [7]. Similarly, Dongola Teaching Hospital and Elobeid Teaching Hospital reported increases in compliance from approximately 50% to over 70-80% after similar structured efforts, highlighting the reproducibility and effectiveness of such interventions across Sudanese healthcare settings [6,9].

The improved documentation in parameters, such as anticipated blood loss (five {10%}-24 {47.8%}, p=0.0001), DVT prophylaxis (0 {0%}-10 {19.6%}, p=0.0027), and postoperative care instructions (0 {0%}-38 {76.1%}, p<0.0001) reflects a broader shift toward safer perioperative care and enhanced communication within surgical teams. These findings parallel those reported in Elobeid, where previously neglected fields like anticipated blood loss and prophylaxis reached 100% compliance after intervention [9]. The clinical significance of documenting these parameters is considerable: failure to record estimated blood loss may hinder postoperative fluid management, while omitted DVT and antibiotic prophylaxis records can directly impact patient safety and surgical site infection risk. These gaps were also evident in audits from Malawi and Jordan University Hospital, further reinforcing the global relevance of such improvements [10,11].

Despite these advances, the study also uncovered persistent weaknesses, particularly in the documentation of the anesthetist’s name (29 {58%}-40 {80.4%}, p=0.0306) and the surgeon’s signature (20 {40%}-32 {63.0%}, p=0.0277). These findings echo trends seen in Wad Madani, where anesthetist details were completely absent from operative records [12]. Such omissions may stem from the entrenched practice of recording certain information, especially anesthetic data and postoperative orders, separately from surgical notes. This fragmentation compromises the completeness of the operative record and may contribute to breakdowns in continuity of care. As observed in the South African audit by Rogers, even experienced surgeons can overlook critical elements, such as wound closure technique and postoperative orders, further emphasizing the need for integrated and standardized recording formats that consolidate all operative details in one document [13].

Another point of comparison is the improvement in documenting surgical findings and complications. Although our audit showed a small increase in operative findings documentation (39 {78%}-46 {91.3%}, p=0.0929), documentation of complications actually declined (26 {52%}-18 {37.0%}, p=0.1585). This result contrasts with findings from India by Javid et al. and the United Kingdom by Cutting et al., where structured feedback and the use of reminders or checklists led to near-complete compliance in these areas [14,15]. The decline in complication reporting may reflect either under-documentation or a genuine reduction in intraoperative issues. However, the former is more likely, particularly in environments where cultural or medicolegal apprehensions discourage transparent reporting. As noted by Babalola et al. in Nigeria, documentation quality is frequently compromised by legibility and completeness issues, both of which may have contributed to the low complication and postoperative care instruction rates in our first cycle [16].

The introduction of standardized proformas and repeated audits has consistently been associated with marked improvements in compliance, as supported by our findings. The use of templates based on the Royal College of Surgeons of England (RCSEng) guidelines has proven effective in a variety of settings, including Arif Memorial Teaching Hospital and Khyber Teaching Hospital in Pakistan, where compliance rose to above 90% postintervention [9,14,17]. However, our audit suggests that templates alone are insufficient without sustained reinforcement through staff training and performance feedback. While our data reflect meaningful gains in documentation of surgical team information (38 {76%}-49 {97.8%}, p=0.0029), type of incision (32 {64%}-42 {84.8%}, p=0.0402), and tissue details (27 {54%}-38 {76.1%}, p=0.0360), achieving the consistent 90-100% compliance levels seen in studies from India and Egypt may require the implementation of additional supports, such as visual aids, EMRs, and checklist-based systems [14,18].

Of particular note is the persistent challenge in documenting postoperative care instructions. Although our study saw an increase from 0 (0%) to 38 (76.1%), this figure still falls short of the 100% compliance seen in Muhammed et al. and Javid et al.’s study [9,14]. Previous studies, including those from Port Sudan and Wad Madani, suggest that this shortfall may be due to traditional practices where postoperative orders are written on separate sheets rather than included in the operative note itself [7,12]. This habit creates a discontinuity in documentation and may result in important instructions being missed by subsequent care teams. Addressing this issue may require not only procedural changes but also cultural and educational shifts within surgical teams, possibly through focused workshops, inclusion of reminders in the proforma, or integration of digital documentation platforms.

The improvement in recording prosthesis usage (seven {14%}-13 {26.1%}, p=0.2113) and extra procedures (10 {20%}-11 {21.7%}, p=1.0000), although limited, is in line with similar results from Dongola, where special attention was given to capturing such technical details [6]. Despite being lower than ideal, these changes demonstrate an emerging awareness of the importance of complete operative records for legal, clinical, and research purposes. In high-income settings, such as the UK and India, audits like those by Cutting et al. and Singh et al. achieved near-total compliance by pairing standardized formats with institutional commitment and digital tools, suggesting that the full potential of documentation improvement efforts can only be realized through comprehensive, system-wide adoption [15,19].

In summary, our findings align with an expanding body of evidence from both Sudanese and international studies that supports the use of structured documentation, regular audits, and targeted training as effective strategies to improve the quality of surgical operative notes. The observed progress in key areas, such as preoperative diagnosis, blood loss estimation, antibiotic prophylaxis, and surgical detail recording, highlights the positive impact of our interventions. However, residual deficits-particularly in postoperative instruction documentation, legibility, and full compliance with all parameters-mirror challenges reported in multiple low-resource settings. These findings reinforce the need for sustained efforts, possibly including electronic health record integration, regular performance review, and cultural shifts in documentation behavior, to achieve and maintain comprehensive, accurate, and high-standard surgical records.

Limitations

This audit has several limitations that should be acknowledged. Firstly, the study was conducted at a single institution with a relatively small sample size (n=50 per cycle), which may limit the generalizability of the findings to other hospitals or settings with different surgical practices. Secondly, the assessment relied on documentation review rather than direct observation of intraoperative events, raising the possibility that some procedures or care elements were performed but not recorded. Thirdly, although efforts were made to ensure consistent assessment, the possibility of assessor bias cannot be fully excluded, particularly in the subjective evaluation of parameters, such as operative findings or tissue details. Additionally, the follow-up period postintervention was short, and the sustainability of improvements over time remains uncertain. Finally, this study did not assess the clinical impact of improved documentation (e.g., on patient outcomes or complication rates), which could be a valuable area for future research. Despite these limitations, the audit offers important insights into the value of structured proformas, training, and regular feedback in enhancing surgical documentation quality in low-resource settings.

Furthermore, the context-specific nature of this intervention means that while the methodology and structured approach are replicable, the outcomes may differ in other countries or even within different hospitals in the region. Variations in institutional policies, staff training levels, and resource availability could influence the effectiveness of similar interventions. Future research should aim to validate these findings through multicenter audits or assess the integration of digital documentation platforms to improve sustainability and generalizability.

## Conclusions

This audit demonstrates that structured interventions, including the implementation of a standardized format and targeted staff education, can significantly enhance the quality and completeness of surgical operative note documentation. Marked improvements were observed across key parameters, particularly in areas critical to patient safety and perioperative communication, such as patient identification, procedure type, blood loss estimation, and postoperative care instructions. However, persistent gaps, especially in documenting anesthetist details, complications, and postoperative plans, underscore the need for continued reinforcement. Practical next steps include appointing documentation champions within surgical teams, conducting regular audits, and maintaining ongoing training sessions to sustain compliance and drive cultural change. These strategies are particularly important in low-resource settings where electronic documentation systems may not be feasible.

## References

[1] Lefter LP, Walker SR, Dewhurst F, Turner RW (2008). An audit of operative notes: facts and ways to improve. ANZ J Surg.

[2] Okoli CM, Adetula UE (2024). Audit of post-operative notes in a Nigerian tertiary hospital: a comparison against the Royal College of Surgeons of England’s Good Surgical Practice Guidelines. Int J Med Health Dev.

[3] Bozbiyik O, Makay O, Ozdemir M, Goktepe B, Ersin S (2020). Improving the quality of operation notes: Effect of using proforma, audit and education sessions. Asian J Surg.

[4] Ibrahim HM, Zeineldeen AA, Alameen MA (2024). Enhancing surgical operative note standards at Doka Hospital, Sudan: a clinical audit. Cureus.

[5] AbouElseoud AM, Gaballah AM (2024). Improving the quality of operation notes: effect of using audits and education sessions. J Adv Med Med Res.

[6] Bakheet OE, Muhammed A, Mohamed A (2024). Evaluation and improvement of the quality of surgical operative notes in the Department of General Surgery at Dongola Teaching Hospital, Sudan. Cureus.

[7] Abdelbagi AY, Muhammed A, Elnour MA (2024). Evaluating and improving the quality of surgical operative notes at the Port Sudan Teaching Hospital. Cureus.

[8] Royal College of Surgeons of England: standards and guidance. Royal College of Surgeons of England.

[9] Muhammed A, Mohamed AM, Ibrahim AF (2025). Evaluating and improving the quality of surgical operative notes at Elobeid Teaching Hospital: a two-phase audit. Cureus.

[10] Alqudah M, Aloqaily M, Rabadi A (2022). The value of auditing surgical records in a tertiary hospital setting. Cureus.

[11] Nyamulani N, Mulwafu W (2018). The quality of hand-written operative notes in a surgical unit at Queen Elizabeth Central Hospital (QECH), Malawi: a prospective completed audit loop study. Malawi Med J.

[12] Mohamed A, Abdalla M (2024). A study in Wad Madani, Sudan: are we documenting operation notes effectively?. Cureus.

[13] Rogers AD (2008). The quality of operative notes at a general surgery unit. S Afr Med J.

[14] Javid M, Swaminathan SP, Jebasingh AV, Velayutham M, Mani R (2020). A prospective closed loop audit on the quality of the operative notes in a general surgical unit in a quaternary care centre. Int J Surg.

[15] Cutting J, Hossain T, Maude K (2014). Quality of operation note documentation in general surgical patients: re-audit results. Int J Surg.

[16] Babalola RN, Olasehinde O, Sowande OA (2016). An audit of the quality of surgical operation notes in a Nigerian Teaching Hospital. East Cent Afr J Surg.

[17] Nzenza TC, Manning T, Ngweso S, Perera M, Sengupta S, Bolton D, Lawrentschuk N (2019). Quality of handwritten surgical operative notes from surgical trainees: a noteworthy issue. ANZ J Surg.

[18] Hassan AG, Elqaffas EO, Elbouridy AM, Shawky MM, El-Fayoumi TA (2023). A closed-loop clinical audit of surgical documentation of inpatient records at a tertiary level hospital in egypt. Cureus.

[19] Singh R, Chauhan R, Anwar S (2012). Improving the quality of general surgical operation notes in accordance with the Royal College of Surgeons guidelines: a prospective completed audit loop study. J Eval Clin Pract.

